# Analysis of spatio-temporal evolution and influencing factors of land dividends in China

**DOI:** 10.1371/journal.pone.0309786

**Published:** 2024-11-08

**Authors:** Yao Wang, Jiahui Ling

**Affiliations:** 1 School of Information Engineering, Southwest University of Science and Technology, Mianyang, Sichuan, China; 2 School of Economics and Management, Tongji University, Shanghai, China; Tallinn University of Technology School of Engineering: Tallinna Tehnikaulikool Inseneriteaduskond, ESTONIA

## Abstract

This paper explores the classification, formation, measurement, evolution, and influencing factors of land dividends in China.It analyzes the nature of land value dividends and efficiency dividends, examines their spatio-temporal evolution, and investigates influencing factors via a regression model, providing insights for future development. The paper posits that changes in land use patterns primarily contribute to land value dividends, while efficiency dividends stem from improving land use efficiency within existing patterns. Presently, China is transitioning from a phase dominated by land value dividends to one marked by efficiency dividends. In terms of spatio-temporal dynamics, efficiency dividends display a fluctuating upward trajectory, whereas land value dividends and the overall quantity of land dividends demonstrate an initial ascent followed by a gradual decline, with the central region exhibiting the most pronounced changes. The analysis of influencing factors reveals a significant positive correlation between value dividends, the overall quantity of land dividends, and factors such as land transfer and capital investment, while efficiency dividends exhibit a significant positive correlation with urbanization. Consequently, the government should implement measures to sensibly regulate land quantity and layout, promote intensive and efficient land use, activate dormant land resources, and propel the optimization and high-quality development of land dividends.

## 1 Introduction

Land stands as a pivotal input for economic development, a fact underscored by its significant contribution to China’s rapid economic growth since the inception of the People’s Republic of China. At the macro level, land assumes a crucial role as a financing tool, with government-driven land policies hastening the pace of the land economy’s expansion (Long, 2014 [1]; Rithmire,2015 [2]; Chen & Long, 2020 [3]). At the micro level, concerted efforts by the government propel the transition of China’s economy from primary to secondary and tertiary industries. The rise in urbanization and industrialization fuels the demand for urban land, accelerates land transfers in rural areas, and bolsters the state’s capacity for large-scale land acquisition (Whiting,2011 [4]; Wong, 2013 [5]).

Land dividends, as a significant driving force for China’s economic growth and social development, play a multifaceted role in fostering progress. Firstly, serving as a fundamental factor of production, the efficient utilization of diverse land types contributes significantly to enhancing residents’ quality of life (Tian & Fang, 2019 [6]). Secondly, during periods of land use transformation, it facilitates the migration of rural populations to urban areas and non-agricultural sectors, thereby fostering regional urbanization and expediting industrial development (Chen et al., 2015 [7]; Luo, 2011 [8]); Thirdly, through the implementation of land policies such as reducing construction land and redeveloping low-efficiency land, the government enhances investment in specific areas, intensifies land use, optimizes land utilization patterns, and refines industrial spatial structures (Verburg et al., 2009 [9]). This not only enhances unit land economic revenue but also ensures ecological environmental optimization (Zhang et al., 2018 [10]; Yan et al., 2010 [11]).

The concept of land dividends, as defined in this paper, encompasses the economic benefits derived from both land use change and improvements in land use efficiency. This definition underscores that land serves as both capital, directly increasing value through land use change (referred to as value-added land dividends), and as a factor indirectly enhancing value through improved land use efficiency (referred to as land efficiency dividends). While land use change remains the primary driver of land dividends, enhancing the efficiency of land resource allocation stands as a crucial means of sustaining them. In accordance with the Land Administration Law of the People’s Republic of China, provinces and cities nationwide are mandated to achieve equalization in the quantity and quality of arable land while protecting the arable land red line, ensuring a balance between arable land occupation and compensation. Additionally, urban construction land totals are regulated by land use plans, with strict controls on the scale of new construction land in all regions.

As a critical natural resource, land faces significant pressure from both local government demand for construction land and environmental protection needs. For instance, in developed areas like Shanghai and Jiangsu, construction land scales have nearly reached saturation, with value-added land dividends approaching regional limits. Addressing this necessitates the formulation of appropriate land policies to accelerate the transformation and upgrading of land use structures, alleviating regional economic imbalances, low production efficiency, and environmental pollution (Pannell, 2011 [12]). The innovation of this paper mainly includes three aspects: 1. The establishment of a land dividend classification framework. This paper defines the connotation of land dividends more comprehensively. Land dividends not only include value-added land dividends generated in land use change but also land efficiency dividends generated in land use efficiency improvement. On this basis, the paper explores the formation mechanism of different types of dividends. 2. Calculation and spatio-temporal evolution of land dividends. Based on the provincial and municipal data in China, the paper estimates the amount of value-added land dividends, land efficiency dividends, and total land dividends, and explores the spatio-temporal evolution of land dividends in China. 3. Analysis of influencing factors of land dividends. Based on the estimated results of land dividends in various regions, econometric models are constructed to find out the main factors affecting land dividends, and policy suggestions are put forward based on the models. All of these are conducive to the Chinese government to formulate land policies with long-term benefits ensuring the growth momentum of land dividends.

## 2 Literature review

The land dividend is one of the development dividends in China. Scholars have yet to reach a consensus on the definition of "land dividend". Most scholars believe that land dividend is the appreciation of land wealth in land capitalization (Tang, 2009 [13]; Gao, 2011 [14]; Zhang, 2013 [15]), that is, the value-added part generated in land use change (Wei, 2014 [16]). Zhang (2013) [15] employed the Solow model to demonstrate the generation of land dividends during the conversion of rural collective land into urban state-owned land. These dividends stemmed from the economic benefits arising from changes in land use during land acquisition. A small number of scholars define land dividend as the maximization of economic benefits based on equal “marginal income” generated by land resources in various uses, which can be obtained by improving the reasonable allocation level of land resources and establishing state control and development of various land uses (Mao et al., 2008 [17]; Dang, 2013 [18]; Dang, 2015 [19]).

Zhang Guanghui (2013) [20] asserts that land dividends do manifest during the transition from collective ownership to state ownership in China. He analyzes the distribution of land dividends among different stakeholders, mainly focusing on the two main bodies of farmers (or village committees) and local governments. The compensation for national land acquisition mainly includes three aspects: compensation for cultivated land loss, resettlement subsidies, and agricultural product loss compensation. All these values are calculated according to the agricultural output value before land acquisition, without considering the future market value of the land. In terms of compensation distribution ratio, the share of economic compensation received by land contractors (farmers) in land dividend distribution is relatively low, generally obtaining no more than 20% of the land share (Zhu & Tang, 2013 [21]; Zhang & Wei, 2015 [22]). Compensation distribution can be one-time cash and pension compensation. One-time cash compensation refers to compensation paid once by the state to villagers who lose their land at a price lower than the market price (Cui et al., 2015 [23]; Rithmire, 2015 [1]). For most rural farmers, land serves as a fundamental pillar of economic production, a vital source of livelihood, and a form of social security (Cai, 2016 [24]; Suranga et al., 2017 [25]. However, the significance of land as an insurance mechanism is often overlooked when calculating one-time cash compensation. This oversight compromises the long-term livelihoods of land-lost farmers while prioritizing short-term economic gains, thereby depriving them of future economic welfare (Guo, 2001 [26]; Heurlin, 2016 [27]; Mattingly, 2016 [28]). At present, the government’s compensation for land loss of rural farmers is mainly realized by providing social welfare for villagers, usually in the form of old-age insurance, which guarantees that eligible villagers can receive lifelong pensions (Cai, 2016 [23]).

To sum up, research on land dividends remains relatively limited worldwide, leaving significant room for improvement. First, the definition of land dividends is not well established. Current studies primarily focus on land dividends arising from land ownership transfers, often overlooking the dividends generated through the efficient allocation of land resources without changing ownership. This includes scenarios such as land use control, the reduction of construction land, and other phases where land efficiency dividends play a crucial role yet receive less attention in existing literature(Huang et al., 2019 [29]). Secondly, the nature of the formation of land dividends is still unclear. At present, the attention on land dividends mainly focuses on the distribution ratio or distribution form of value-added land dividends among different stakeholders, and there is no relevant research on the formation and existing forms or different types of land dividends. Thirdly, the temporal and spatial evolution characteristics of land dividends are still unclear. The foundation of land dividends lies in value-added land dividends, while land efficiency dividends represent the primary avenue for future growth in land dividends. However, current research has not quantified the extent of existing land dividends from available data, making it challenging to illustrate the spatio-temporal evolution characteristics of land dividends across diverse regions. Finally, there are no papers to analyze the factors affecting land dividends at present.

In this context, the paper explores the nature of land dividend formation from the perspectives of value-added and efficiency enhancement and uses the provincial and municipal data in China to measure the total amount of value-added land dividends, land efficiency dividends, and land dividends. The paper analyzes the spatio-temporal evolution of land dividends and further finds out the main factors influencing land dividends from an empirical perspective. Compared to the existing literature, this paper makes a relatively comprehensive and in-depth interpretation of land dividends from the aspects of “classification, formation, calculation, evolution and influence” of land dividends to enrich the existing land dividend theory.

## 3 Classification and calculation of land dividends

### 3.1 Classification of land dividends

#### 3.1.1 Value-added land dividends

The formation of value-added land dividends mainly stems from the difference in the organic composition of capital among different types of land resources, that is, the land value increment generated in land use changes from one to a more economically rewarding use. Different types of land exert varying impacts and contributions to economic growth. Generally, industrial construction land contributes significantly more to land economic dividends than agricultural land (Lichtenberg & Ding, 2009 [30]), and agricultural land is higher in that contribution than unused land. This difference in contribution is particularly obvious in the phases of urban construction and transformation. Value-added land dividends mainly exist in two forms: the conversion of agricultural land to industrial construction use and the transformation of unused land into agricultural land. The conversion of agricultural land into industrial construction land stands as the primary form of value-added land dividends.

Consider the case of agricultural land transitioning to industrial construction land, where the enhancement of land development rights becomes particularly pronounced. This enhancement is evident not only in the increased economic compensation for farmers—who collectively benefit from expanded land value-added income programs—but also in its role in driving national industrialization and urbanization, reducing income inequality, and fostering urban-rural economic development. The strengthening of land development rights reflects broader societal progress. Furthermore, the increased valuation of land development rights by both government and market mechanisms in land allocation and revenue distribution helps safeguard the rights and interests of farmers in rural areas. This, in turn, boosts overall land use efficiency, thereby advancing the state’s objectives of rejuvenating underutilized land and promoting ecological protection and restoration in a coordinated and sustainable manner.

During land use transitions, land prices encapsulate not only the value of natural resources but also the embodied human labor invested in the land, primarily manifested monetarily as land capital prices. Land use rights and the labor invested in the land are concomitantly transferred with capital, implying that the realized labor invested in the land is simultaneously monetized. However, due to natural conditions, agricultural technology, and other factors, achieving a significant increase in agricultural output in the short term post-transition is challenging. Nonetheless, the transition from agricultural to industrial construction land enhances resource allocation efficiency, leading to a significant increase in land revenue.

For instance, in Shanghai, the arable land compensation system was initiated in 2013, with the implementation of the "balance of arable land occupation and compensation" principle while converting arable land to non-agricultural use, concurrently replenishing arable land with equivalent quantity and quality (Ma & Liu, 2016 [31]; Liu et al., 2017 [32]). Simultaneously, agricultural land, low-efficiency construction land, and unused land underwent reorganization and reclamation to obtain arable land balance indicators, further alleviating the tension between economic development and agricultural land protection. As the land economy transitions from sheer quantitative advantage to comprehensive efficiency advantage, the government places greater emphasis on fairness and justice in development, realizing mutual benefits derived from societal development, constructing a social security mechanism for landless farmers, and fostering the evolution of land dividends from value-added land dividends to land efficiency dividends.

## 3.1.2 Land efficiency dividends

As the spatial planning system of various provinces and cities undergoes gradual enhancement, the expansion of urban development boundaries is no longer viable beyond the ecological protection red line. Permanent basic farmland and urban development boundary red lines are established within the planning system. The elucidation of functional layout and planning for basic farmland and ecological land, along with the optimization of internal spatial structures of cities and towns, emerges as new considerations for the future. The crux of land efficiency dividend formation lies in the government’s stringent reduction of transferable land and expedited transformation to enhance land use efficiency. Enhanced land use efficiency facilitates the optimization of current land use patterns, adjustment of industrial location layouts, and optimization of industrial spatial agglomeration structures. Whether land ownership is transferred or retained, the majority of landowners tend to intensify land use, thereby augmenting their benefits in most cases. However, due to land resource scarcity and land supply monopolies, land capital exhibits attributes akin to financial products, susceptible to speculation and driving up land prices. Entrepreneurs, recognizing the potential for economic profit, improve land use efficiency to garner increased economic gains, resulting in a steady rise in land efficiency dividends with enhanced land use efficiency. In the context of an open-market economy, efficient land use not only stimulates domestic investment and purchasing power but also generates a spillover effect of land economic dividends through products manufactured by enterprises traded globally via international trade and other channels.

With economic development and social progress, the expansion of construction land in many Chinese cities nears its limit. Emphasis on ecological environment and resource and environmental carrying capacities has gradually become a focal point of China’s economic development. Accordingly, the mode of land dividend formation must adapt to economic development changes. Land efficiency dividends primarily manifest in three forms: land acquisition dividends, construction land reduction dividends, and land secondary development dividends. Land acquisition dividends arise from state acquisition of land during the transformation of collective land into state-owned land. For instance, the transfer of construction land ownership mainly occurs in the land secondary transaction market, transitioning ownership from collective to state-owned. Following construction land ownership transfer in the land market, the government enhances regional infrastructure to attract potential enterprises. Rational planning fosters intensive and efficient land use, facilitating the formation and growth of land economic dividends. Construction land reduction dividends entail transferring and optimizing construction land allocation, reducing quantity while increasing efficiency, and enhancing construction land utilization intensity by compressing and downsizing existing construction land to achieve efficiency goals. Land secondary development dividends stem from effective land use through planning management, urban renewal, and other methods to rejuvenate land resources amid issues like scattered layouts, low plot ratios, and inefficient use.

## 3.1.3 Relationship between value-added land dividends and land efficiency dividends

With the decrease of transferable land, land use encounters challenges in transitioning from agricultural to construction purposes. On one hand, such transitions necessitate stringent government approval, while on the other hand, the quantity of land convertible to state-owned construction is severely restricted. Consequently, alterations in land use and ownership give rise to the emergence of "value-added dividends" and "efficiency dividends". The formation nature of value-added land dividends and land efficiency dividends is shown in Table 1.

**Table 1:** Nature of land dividends formation.

Types of Land Dividends	The Nature of Land Dividends Formation
Value-added Land Dividends	Different types of land have different organic compositions of capital
Land Efficiency Dividends	Increased land input intensity drives land use efficiency

The obvious difference between value-added land dividends and land efficiency dividends is that the value-added land dividend is mainly a leaping change process, while the land efficiency dividend is a gradual change process. Land efficiency dividend is a supplement to the limited growth of value-added land dividends, which ensures the long-term and sustainable growth of land dividends.

The prerequisite for realizing land efficiency dividends lies in the effective utilization of land resources. While the decline in value-added land dividends follows an irreversible trend, the potential for development in land efficiency dividends is vast. Traditional land use changes may expedite the conversion of agricultural land to construction land, thereby boosting regional output in the short term. However, achieving sustainable output growth in the long term proves challenging. China’s value-added land dividends are gradually waning. To continue reaping the economic, social, and ecological benefits bestowed by land dividends, it becomes imperative to further enhance land use efficiency and transition the source of land dividends from value-added to efficiency dividends.

### 3.2 Calculation of land dividends

Land serves as the foundation of economic activities. Assuming all other factors remain constant, the economic dividends arising from land primarily manifest in the form of enhanced economic growth resulting from the judicious utilization of various land types. This enhancement is notably reflected in three main aspects: firstly, maintaining the land’s original use yields certain economic benefits for the landowner; secondly, changes in land use can lead to short-term regional economic surges (e.g., transitioning agricultural land to industrial construction purposes); and thirdly, efficient land utilization fosters additional economic gains, building upon existing benefits and facilitating further economic expansion.

There are three primary categories of land use: unused land, agricultural land, and construction land. However, due to significant variations in output value per unit area between industrial construction and commercial service land, the paper distinguishes between two types of construction land: industrial construction and commercial service land. Consequently, the four main types of land use considered are: unused land, agricultural land, industrial construction land, and commercial service land. Assumptions: (1) With the exception of unused land, agricultural, industrial construction, and commercial service land are all profit-generating; (2) The quantity of unused land remains relatively stable over the research period and does not change within the specified timeframe; (3) The expansion of industrial construction land occurs primarily through the transformation of agricultural land, while the growth of commercial service land is predominantly observed through the conversion of industrial construction land.

As the economy’s land use structure undergoes rapid changes, urban industrialization and urbanization processes accelerate, leading to a larger volume of value-added land dividends. However, over time, the pace of land use conversion gradually slows, resulting in a decline in the rate of structural change and limiting the expansion of value-added land dividends. Consequently, the per-unit area utility of non-agricultural land indirectly increases, motivating landowners to enhance economic output per unit area by improving land use efficiency. Some scholars refer to this as the enhancement of land use value and revenue, termed as the value of land development rights [33]. The deceleration of land conversion trends compels entrepreneurs and landowners to intensify land use through increased investment, aiming to attain certain levels of land efficiency dividends. Amidst land appreciation, rapid changes in the economy’s land structure spur urban industrialization and urbanization. Conversely, with improvements in land efficiency, the economy’s land use structure experiences minimal change or remains static, with the city’s economic development manifested through enhanced land use efficiency.

The rise in land dividends signifies either an rise in the frequency of land use alterations or an enhancement in the value of land development rights, contributing to an economy marked by rapid growth. Assuming that improvements in land use efficiency correlate with enhancements in land use value, the true essence of land dividends lies in the economic growth effect resulting from changes in land use structure and the value of land development rights amidst urbanization and industrialization. Land use structure changes primarily manifest through alterations in land use, taking the form of either the conversion of agricultural land into industrial construction land or the transformation of industrial construction land into commercial service land. Meanwhile, the enhancement of land development rights value is evidenced by land transitioning to more economically viable uses, such as the conversion of agricultural land into industrial construction land. In contexts of land appreciation, the magnitude of land dividends hinges on the extent of land use change, with its essence embodied in the economic growth effect stemming from alterations in land use structure. Conversely, amid land efficiency improvements, where the land use structure remains static or undergoes minimal change, the essence of land dividends manifests in the economic growth effect catalyzed by the escalation in the value of land development rights. In summary, the magnitude of land dividends can be expressed as follows:

Land Dividends = Value of Land Development Rights * land area changed for use

There is a significant correlation between the quantity of land dividends and changes in land use structure and the value of land development rights. On a macro level, the volume of land dividends is directly linked to the conversion of agricultural land to industrial construction land, industrial construction land to commercial land, and similar transitions. At a micro level, when the government imposes strict regulations on land use conversion due to imbalances in the land market, there is a notable reduction in the number of land conversions. Paradoxically, this restriction inadvertently triggers an upsurge in land use prices.

The quantities of different types of land dividends are expressed as:

1. Value-added land dividends: Represent the land dividends resulting from the difference between the land change area of the current year and that of the previous year, with the initial year within the study area serving as the baseline.
2. Land efficiency dividends: Denote the land dividends arising from the disparity between the land change area of the current year and that of the previous year, with the initial year within the study area serving as the reference point.
3. Total real land dividends: The aggregate sum of both value-added land dividends and land efficiency dividends.

## 4 Model setting and variable selection

### 4.1 Variable selection

#### 4.1.1 Selection of variables for land dividends calculation

1. Land Development Rights Valuation
  Land development rights entail the privilege of profiting from the conversion of agricultural or other land types into those with comparatively higher output. Drawing on the methodology proposed by Tang (2019) [33], the development right value of agricultural land relative to industrial construction land (P1) is expressed as the increase in land value when agricultural land is converted into industrial construction land. P1 = Value of industrial construction land—value of agricultural land. The development rights value for industrial construction land relative to commercial service land (P2) is depicted as the increase in land value when a unit of industrial construction land is transformed into commercial service land, P2 = commercial service land value—industrial construction land value. The value per unit of agricultural land is calculated as the ratio of primary industry output value to agricultural land area, while the value per unit of industrial construction land and commercial service land are computed as the ratios of secondary and tertiary industry output values to their respective land areas.
2. Land Conversion Area Measurement
  The land conversion area encompasses the quantity of agricultural land converted into construction land and industrial construction land transformed into commercial land. As per the assumption in section 3.2, changes in agricultural land can be determined by cross-referencing data on agricultural land from land construction approvals and agricultural land increases for development and reclamation. Similarly, changes in industrial construction land and commercial service land can be assessed by collating data on construction land approvals and land consolidation, development, and reclamation.

## 4.1.2 Selection of variables for influencing factor analysis

1. Government level: Government land transfer policies have hastened the formation and expansion of land dividends. Since the inception of reform and opening up, China has implemented policies aimed at accelerating urban and rural development, optimizing regional industrial structures, and facilitating production flow. These measures have expedited urbanization across provinces and cities, thus ensuring the growth of land dividends. Moreover, government restrictions on agricultural land transfer indices have reduced available land for transfer, implicitly enhancing land use efficiency among entrepreneurs. This paper utilizes district-level land transfer revenue (lnlandsalesrevenu) as a proxy variable for government land transfer behavior, and the proportion of urban and rural employment in the region’s total population (lnemploymentrate) to gauge urbanization.
2. Market level: Changes in land use structure influence land value. Government development of lands or related infrastructure enhancements such as roads, water, electricity, and green spaces, as well as amenities like schools and hospitals, contribute to improved and value-added land dividends. Regional fixed asset investments significantly drive regional output. This study employs capital input (lnasset) as a market-level proxy variable, with fixed asset investment serving as the measurement.
3. Social level: Residents’ focus on social welfare and ecological environments has diluted their pursuit of land economic dividends. Land serves as the fundamental livelihood and material support for rural residents, affecting their welfare. The slowdown in converting agricultural land to industrial construction land contributes to rural residents’ welfare. This paper adopts social welfare level (lndisposableincome) as the social-level proxy variable, measured by rural residents’ disposable income.
4. Enterprise level: Labor force and technological innovation are essential for unleashing land dividends. China’s economy is currently in a phase of medium-to-high-speed development, driving adjustments in regional industrial structures and human resource stocks and quality. Technology enhances land use efficiency and resource allocation while fostering high-quality economic development. Labor wages and technological innovation serve as firm-level proxy variables, with average employee salary (lnsalary) and number of patent applications (lnpatent) chosen for measurement.
5. International level: International trade creates opportunities for land dividend formation. China’s annual exports include resources and land production factors. Generally, higher total import-export volumes correlate with faster regional development rates. This suggests that development through regional opening up is more conducive to stable development, urbanization, industrialization, and rapid economic growth. This study uses trade import-export level (lntrade) and foreign direct investment level (lnFDI) as international-level proxy variables, measured by total trade import-export volume and FDI, respectively. To mitigate multicollinearity and heteroscedasticity, variables are log-transformed.

To reduce the probability of multicollinearity and heteroscedasticity between variables, the corresponding variables have to be processed with logarithm.

### 4.2 Data source and model setting

Since the implementation of reform and opening up, China’s statistical investigation system has gradually improved, aligning its national accounting system with international standards. Due to the absence of land nature data before the 20th century, this paper analyzes and calculates the amount of land dividends using data from various provinces and cities in China from 1999 to 2017 (excluding the Tibet Autonomous Region, Hong Kong, Macao, and Taiwan). The data primarily sourced from publications such as the "China Statistical Yearbook," "China Land and Resources Statistical Yearbook," "China Land and Resources Yearbook," as well as provincial and municipal statistical yearbooks.

This paper uses the stepwise regression method to build models to verify the impact of each variable on land dividends. The calculation models are shown as follows:

$$lnValueaddeddividends=\alpha_{it}*X_{it}+\varepsilon_{it}$$ (4.1)

$$lnefficiencydividends=\beta_{it}*X_{it}+\xi_{it}$$ (4.2)

$$lntotaldividends=\gamma_{it}*X_{it}+\zeta_{it}$$ (4.3)

Eq 4.1 is used to analyze the factors influencing the value-added land dividends. Eq 4.2 is used to analyze the factors influencing land efficiency dividends. Eq 4.3 is used to analyze the factors influencing the total land dividends. lnValueaddeddividends, lnefficiencydividends, and lntotaldividends represent the logarithmic values of the number of value-added land dividends, the number of land efficiency dividends, and the total amount of land dividends, respectively. X on the right side of the equation is the index variable selected based on theoretical analysis. α, β, and γ represent the corresponding coefficient of the influencing factors of different types of land dividends. If the coefficient is less than 0, it indicates that possible independent variables may have a negative influence on the number of dividends; ε, ξ, and ζ represent the random error term of different equations; i and t respectively represent the region and year.

### 4.3 Spatio-temporal evolution

**Fig 1:**
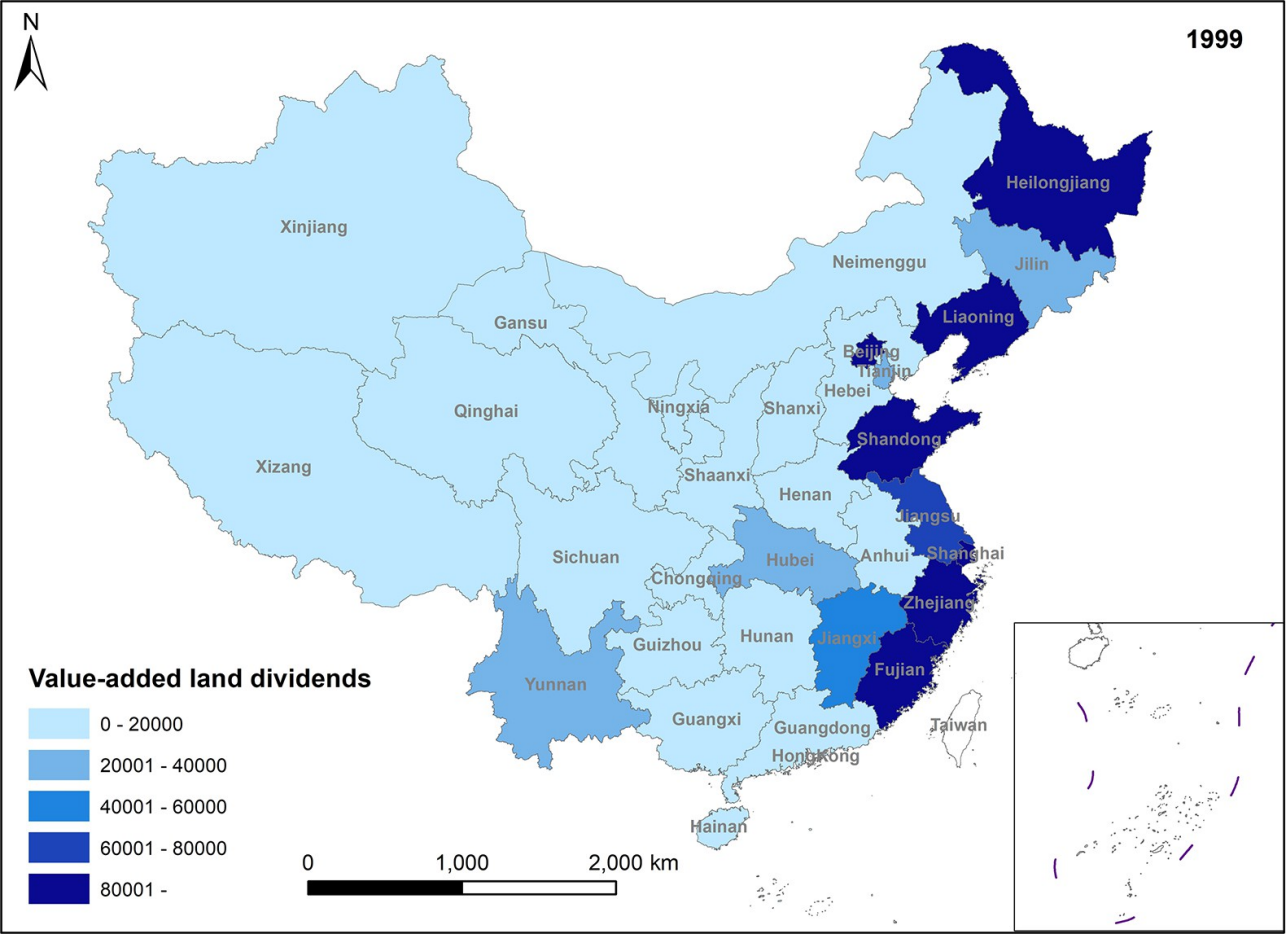
Spatio-temporal evolution trend of value-added land dividends (1999).

**Fig 2:**
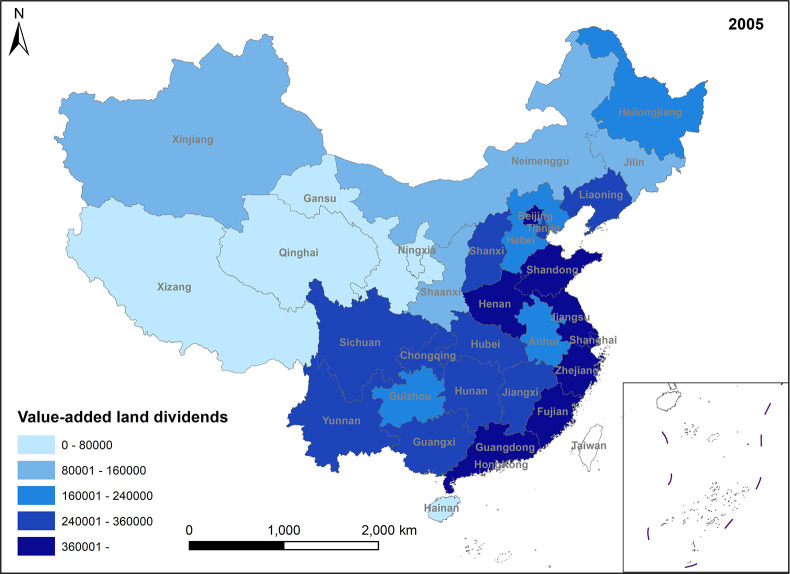
Spatio-temporal evolution trend (2005)-added land dividends (2005).

**Fig 3:**
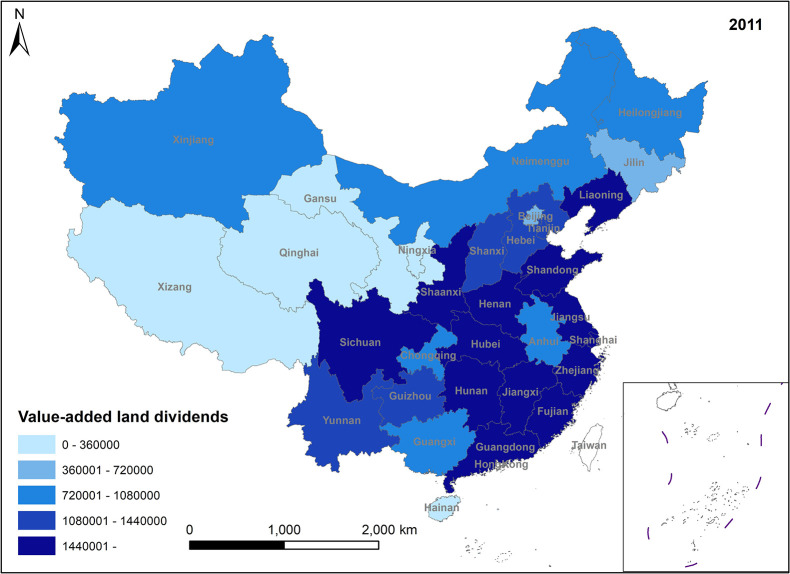
Spatio-temporal evolution trend of value-added land dividends (2011).

Since the 20th century, China’s land dividends have undergone three distinct stages. In the first stage, while maintaining the 1.8 billion-acre red line for arable land, local governments took the lead in transforming the nature of land use. During this period, the rate of land use conversion gradually increased, although the value of land development rights saw only modest growth. Notably, the growth rate of land development rights lagged behind the pace of land use transformation during the first stage. In the second stage, the frequency of land use changes remained high, resulting in a rapid increase in the value of various land development rights. This surge was primarily driven by an imbalance between supply and demand in the land market. In the third stage, characterized by a more stringent national land supply policy, the frequency of land use changes decreased. This reduction further amplified the value of land development rights, sustaining their upward trajectory. Currently, China is transitioning from the second to the third stage, marked by a shift in the dynamics of land dividends. The formation of different types of land dividends is illustrated in Figs 1–3.

**Fig 4:**
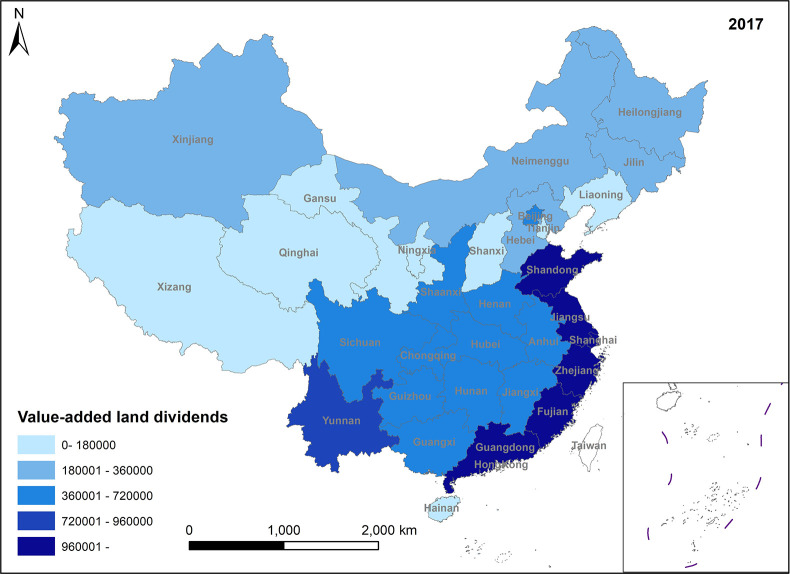
Spatio-temporal evolution trend of value-added land dividends (2017).

In Figs 1–4, the trend of value-added land dividends generally shows an initial increase followed by a decline. The eastern region, including Shandong, Zhejiang, and Shanghai, experiences rapid changes in land use structure, resulting in relatively high levels of value-added land dividends. Similarly, the central region, dominated by Hunan, Hubei, and Jiangxi, as well as the western region, led by Xinjiang and Sichuan, saw their value-added land dividends peak around 2011, followed by a slowdown in growth. The decrease in the number of land use changes is the primary reason for the decline in land appreciation dividends in later periods. This decrease is mainly attributed to several factors: 1) Under the national land tightening policy, when the government needs to expand the area of construction land, it prioritizes converting land with low construction land use efficiency. This approach ensures the efficient use of construction land while avoiding potential social issues associated with land conversion. 2) Agricultural land serves social security and ecological landscape functions, limiting its conversion. Apart from providing economic security for landowners, agricultural land also plays a crucial role in safeguarding the welfare of rural residents and maintaining ecological quality. These functions are difficult to fulfill in the long term with other land use types. Consequently, the government has enacted policies to promote the conversion of inefficient construction land into agricultural and ecological land. 3) Political reasons, such as maintaining the 1.8 billion-acre arable land red line and implementing land use control policies, also restrict land conversion. These policies aim to preserve existing agricultural land and reduce its non-agricultural conversion, indirectly contributing to the gradual increase in land use value.

**Fig 5:**
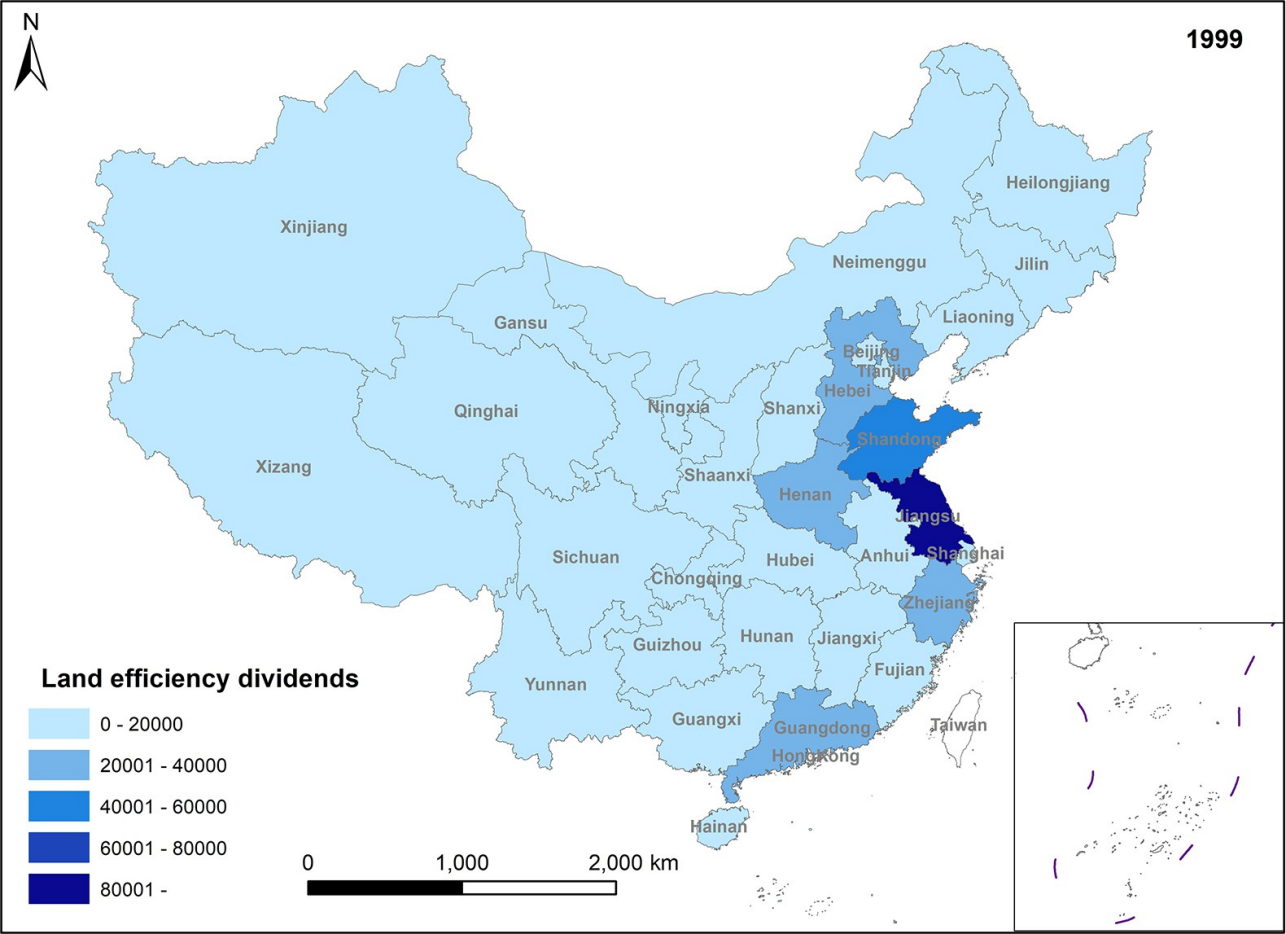
Spatio-temporal evolution trend of land efficiency dividends (1999).

**Fig 6:**
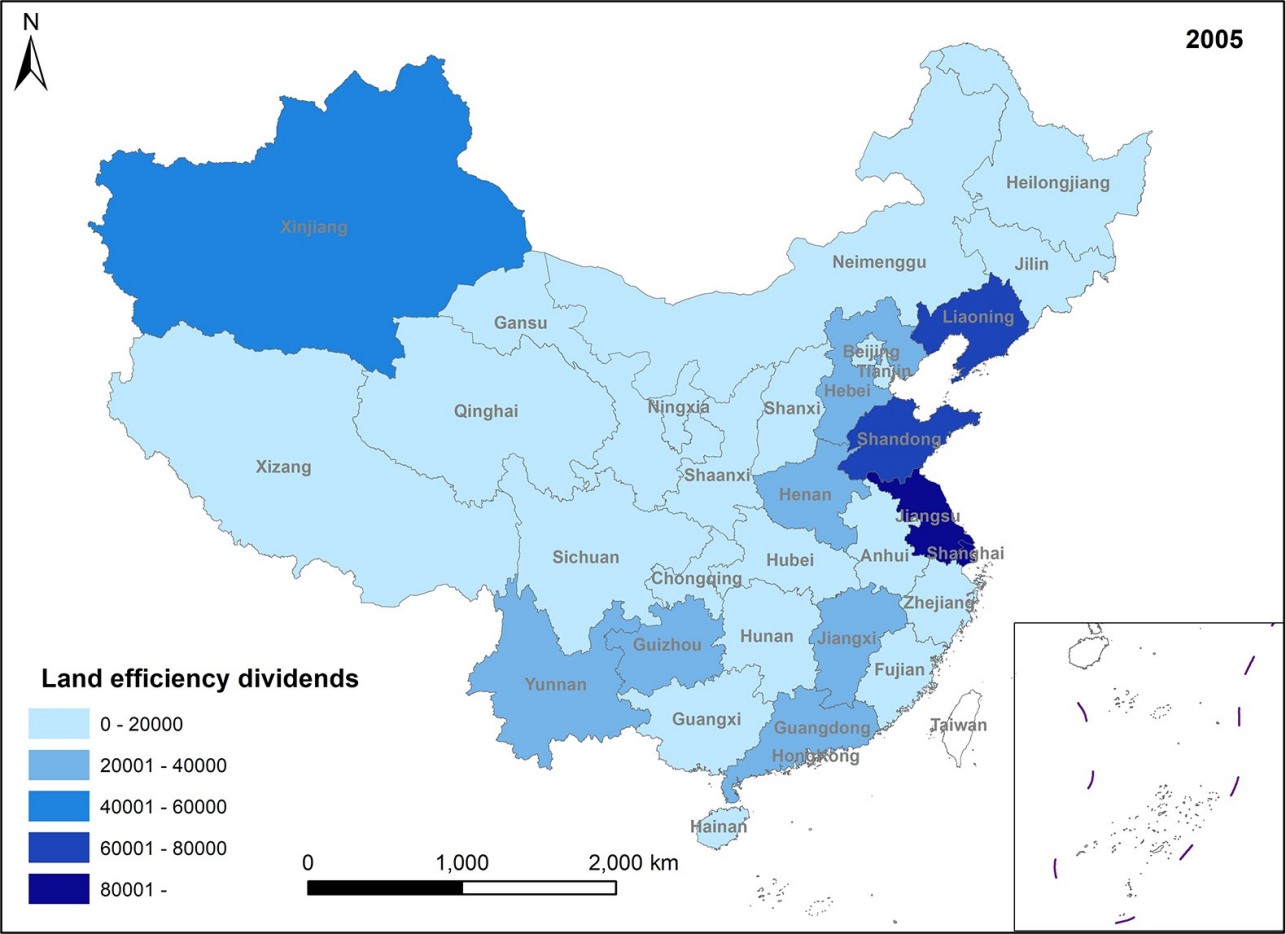
Spatio-temporal evolution trend of land efficiency dividends (2005).

**Fig 7:**
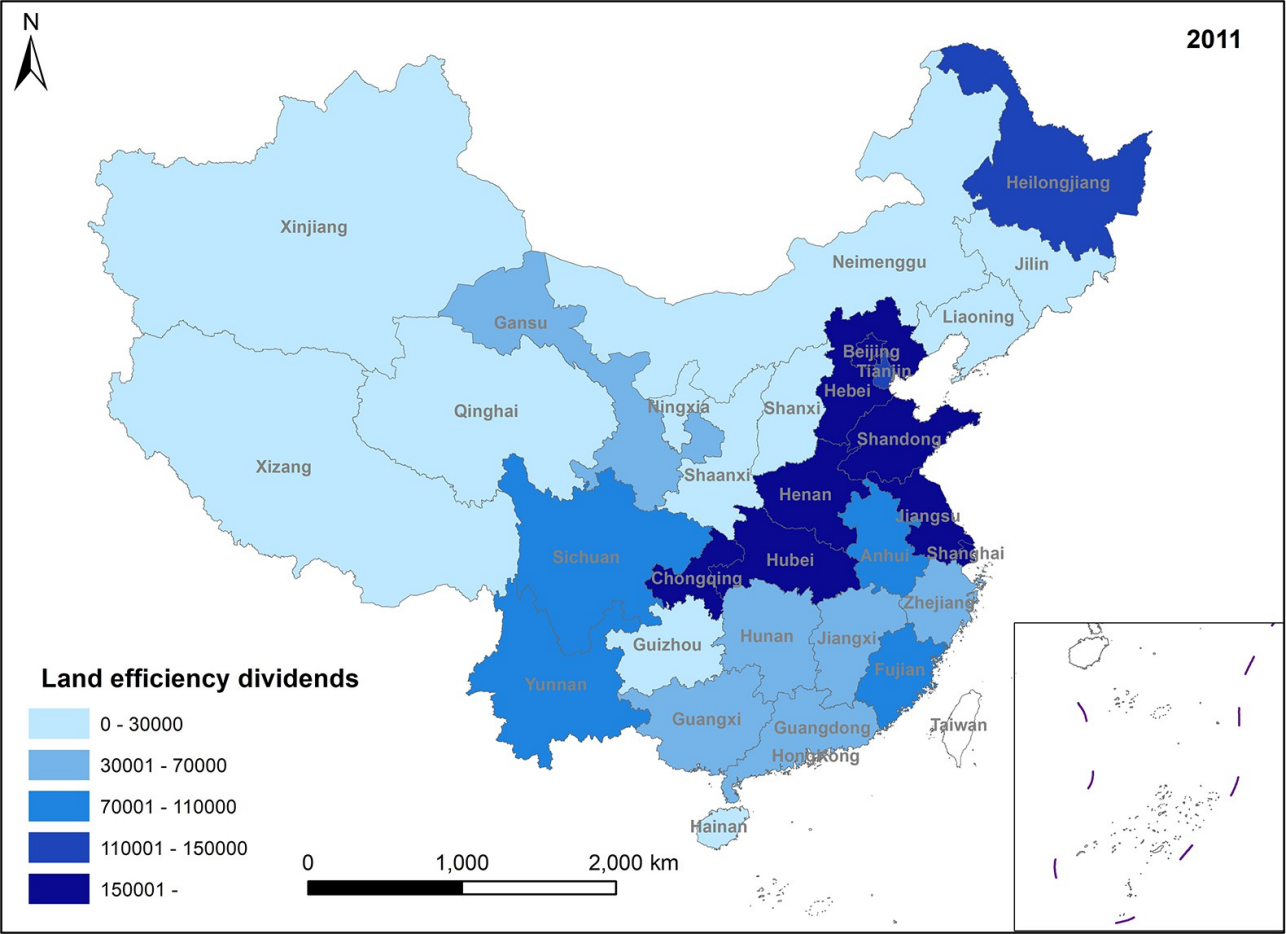
Spatio-temporal evolution trend of land efficiency dividends (2011).

**Fig 8:**
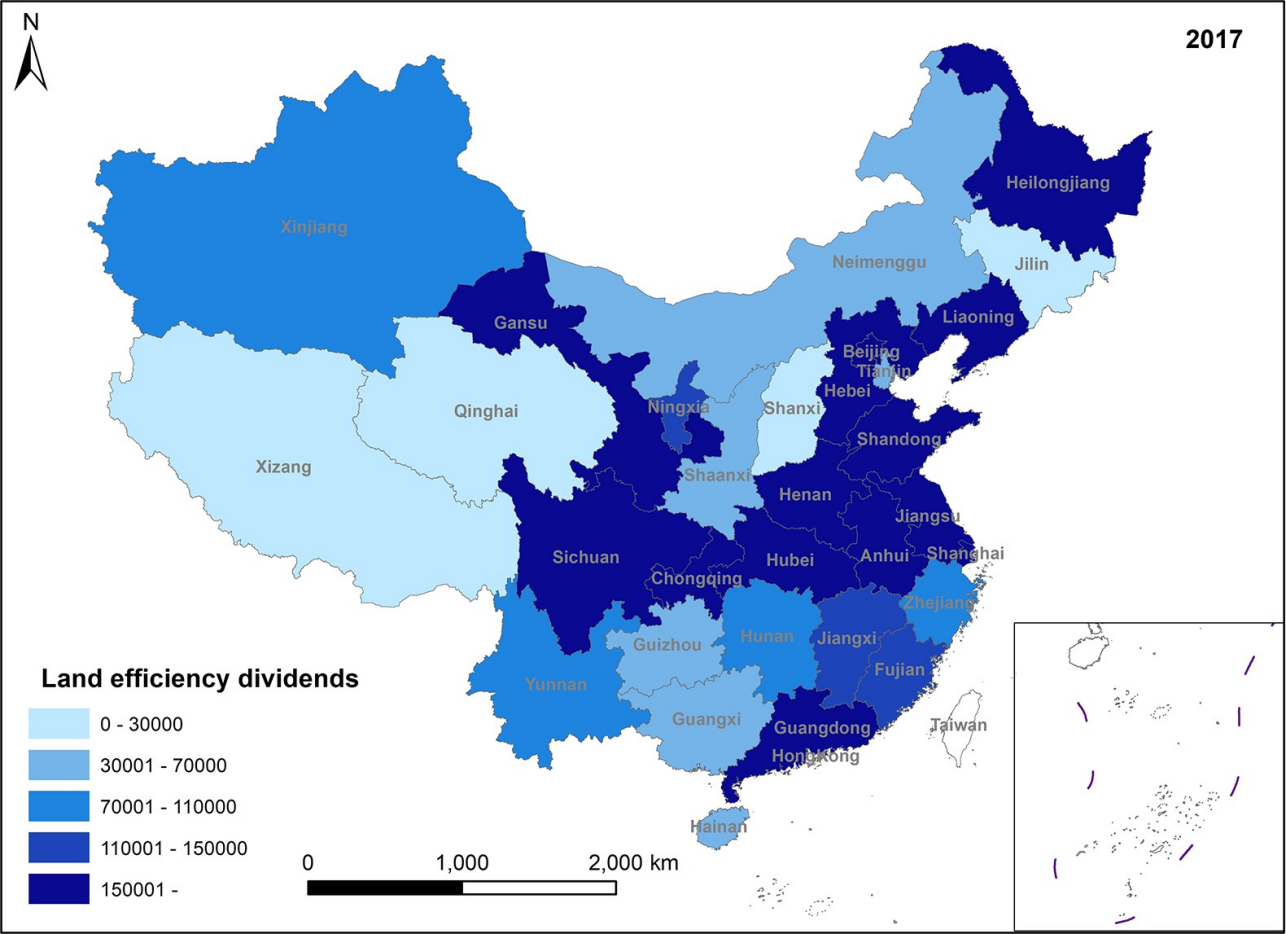
Spatio-temporal evolution trend of land efficiency dividends (2017).

In Figs 5–8, land efficiency dividends show an overall upward trend. The eastern region, represented by Beijing, Tianjin, Guangdong, and Jiangsu, along with the central region, represented by Henan and Hubei, and the western region, represented by Gansu and Sichuan, demonstrate relatively high growth rates. The continuous expansion of total urban construction land and annual additions of new construction land contribute to an extensive land use model, leading to inefficiencies in some land utilization practices. To mitigate issues such as uncontrolled land expansion, the state has introduced pertinent policies. For instance, in 2014, the Ministry of Land and Resources proposed implementing a strategy for total control and reduction of construction land. China’s 13th Five-Year Plan, officially enhancing construction land reduction management in 2015, aims to decrease and recycle all forms of inefficient construction land in suburban areas. In reality, amid escalating demand for urban construction land and comprehensive land use controls, the key to future urban development lies in bolstering the management of both the quantity and intensity of land resources, enhancing land use efficiency, and ensuring a stable rise in land efficiency dividends. Ensuring the judicious growth of land efficiency dividends is paramount. Firstly, it can facilitate the transformation and adjustment of economic and factor structures. Encouraging the development of technologically advanced, low-consumption industries can effectively utilize land factors for development and promote regional spatial optimization and sustainable economic growth. Secondly, under the construction land reduction linkage model, the economy and rational utilization of construction land can be advanced through both new land allocation and the redevelopment of old land and land reclamation. This approach can also facilitate remote allocation of construction land quotas. Furthermore, it addresses issues related to construction renewal and upgrading, thereby enhancing the overall production factor distribution efficiency across the region.

**Fig 9:**
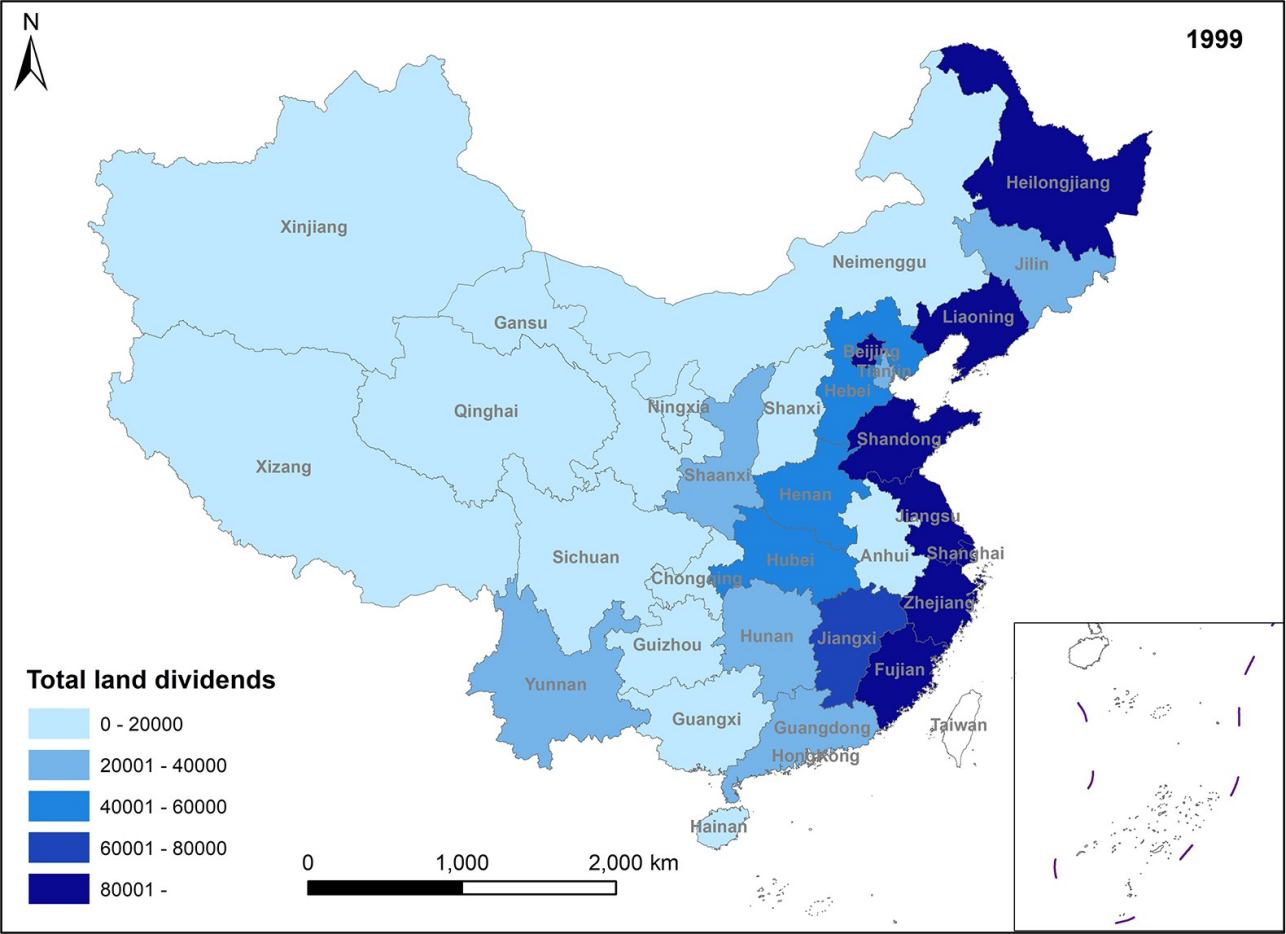
Spatio-temporal evolution trend of total land dividends (1999).

**Fig 10:**
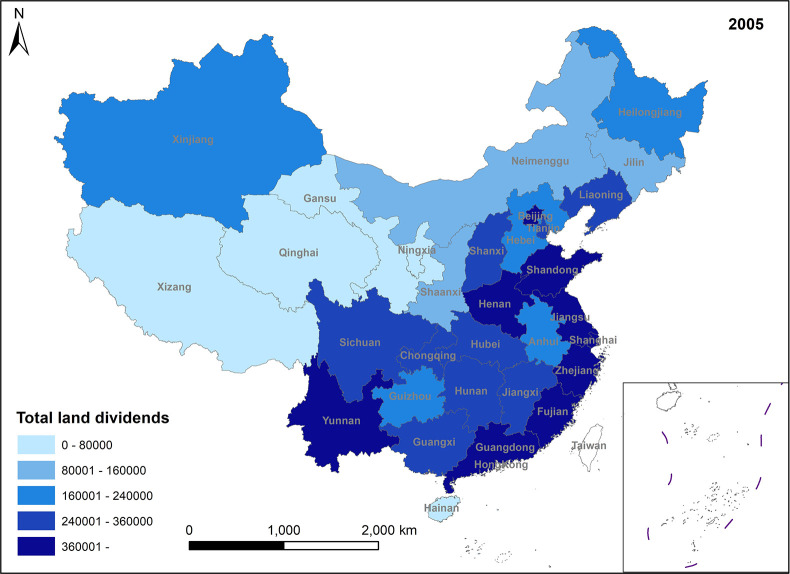
Spatio-temporal evolution trend of total land dividends (2005).

**Fig 11:**
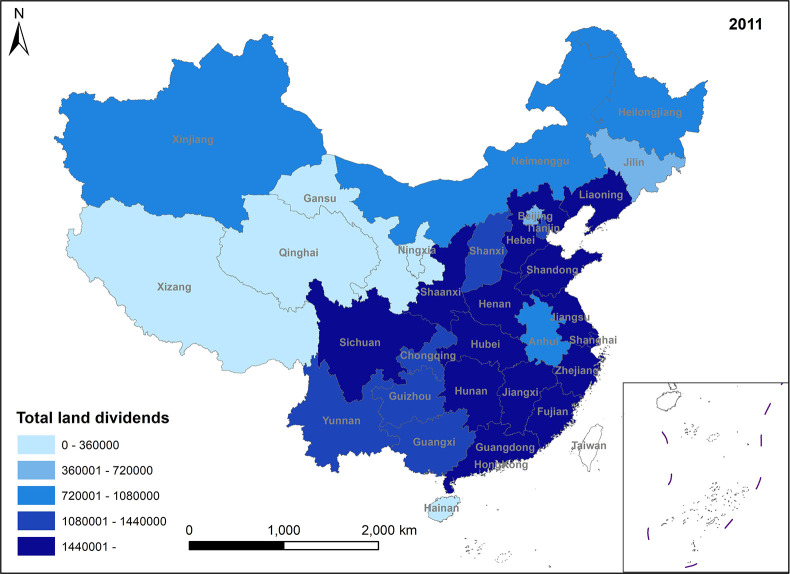
Spatio-temporal evolution trend of total land dividends (2011).

**Fig 12:**
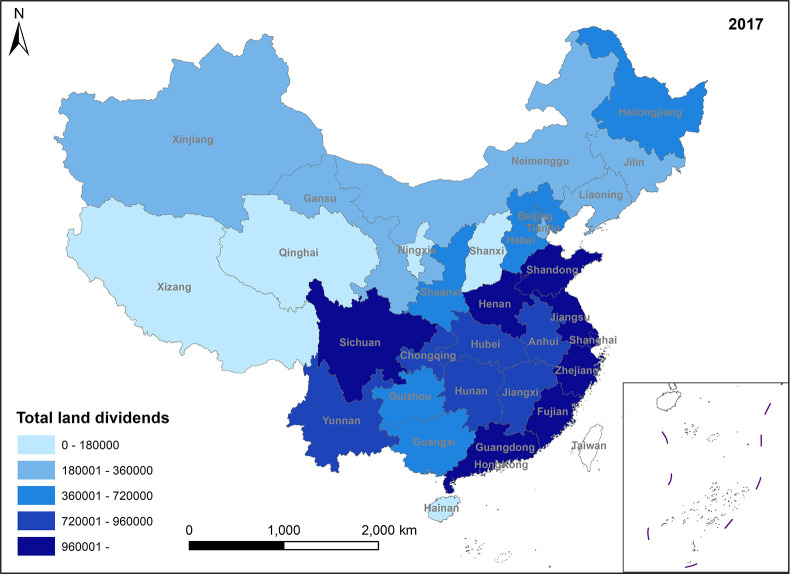
Spatio-temporal evolution trend of total land dividends (2017).

In Figs 9–12, the total land dividend mirrors the fluctuations in value-added land dividends. According to the data, it can be observed that the total amount of land dividend mostly comes from value-added land dividends, indicating that land use transformation is still the main way of land dividend formation.

## 5 Empirical analysis

### 5.1 Descriptive analysis

Before analyzing the influence relationship between variables, it’s essential to grasp their fundamental characteristics. The statistical description of variables is shown in Table 2.

**Table 2:** The descriptive statistical results.

Variables	Number of variables	Mean	Standard deviation	Minimum value	Maximum value
lnValueaddeddividends	588	12.4477	1.8298	5.3046	15.5935
lnefficiencydividends	581	9.6954	2.3666	0.658	14.7764
lntotaldividends	588	12.619	1.7599	5.3158	15.624
lnsalary	589	10.1598	0.7252	8.7103	11.7883
lnlandsalesrevenu	588	7.8898	3.4444	0.1222	16.2757
lnasset	589	8.2056	1.3828	3.9808	10.9188
lndisposableincome	589	9.5463	0.62	8.3749	11.0445
lnpatent	589	8.5122	1.8404	1.9459	12.7149
lntrade	589	14.2644	1.9226	9.1368	18.5083
lnFDI	589	11.746	1.996	0.6931	14.8816
lnemploymentrate	589	0.4662	0.1857	0	0.741

In Table 2, the mean and maximum values of value-added land dividends and total land dividends exhibit marginal disparities, with relatively minor fluctuations. This suggests that, quantitatively, the bulk of the total land dividend originates from value-added land dividends, corroborating the findings in Section 4.3. The logarithmic mean value of land efficiency dividends lags significantly behind that of value-added land dividends, primarily due to two key factors: 1) Historical Context: From the late 19th century to the early 20th century, rapid urbanization and industrialization fueled a sharp increase in land demand. Local authorities, utilizing land supply as a strategic policy tool, expanded infrastructure, attracted investments, and facilitated urban growth, thereby generating substantial value-added dividends. 2) Methodological Considerations: The variation in the value of land development rights within the study area is relatively limited compared to the variation in the area of land use conversion. Consequently, a larger portion of land dividends is derived from the appreciation associated with the scale of land use conversion rather than from increases in land efficiency.

### 5.2 Stepwise regression

Taking the value-added land dividend as an example, to avoid multicollinearity between variables, this paper mainly selects the stepwise regression method to build regression models. The outcomes are depicted in Table 3, where Models (1)—(8) present the regression analysis findings influencing value-added land dividends. It can be seen from the fitting results that the R2 of the equation keeps increasing with the continuous addition of explanatory variables, indicating that the fitting effect of the model is gradually optimized. Therefore, model (8) is selected as the final regression equation affecting value-added land dividends. Similarly, the gradual regression results of land efficiency dividends and total land dividends are shown in models (9) and (10).

**Table 3:** Econometric regression results for different types of land dividends.

Variables	lnValueadded- dividends	lnValueadded- dividends	lnValueadded- dividends	lnValueadded- dividends	lnValueadded dividends	lnValueadded dividends	lnValueadded dividends	lnValueadded dividends	Lnefficiency dividends	Lntotal dividends
	(1)	(2)	(3)	(4)	(5)	(6)	(7)	(8)	(9)	(10)
lnasset	0.943***	0.987***	0.950***	1.175***	1.174***	1.023***	0.825***	0.842***	0.171	0.732***
	(0.109)	(0.112)	(0.113)	(0.116)	(0.116)	(0.123)	(0.129)	(0.129)	(0.247)	(0.115)
lntrade		-0.151	-0.142	-0.190*	-0.184*	-0.170*	-0.155	-0.128	0.112	-0.133
		(0.101)	(0.101)	(0.0977)	(0.1)	(0.0992)	(0.0975)	(0.0977)	(0.187)	(0.0874)
lnpatent			0.00653**	0.00612**	0.00627**	0.00456	0.00481	0.00379	0.00485	0.00381
			(0.0032)	(0.0031)	(0.0031)	(0.0031)	(0.0030)	(0.0031)	(0.0061)	(0.0028)
lndisposableincome				-3.160***	-3.171***	-4.289***	-4.105***	-4.209***	0.144	-2.967***
				(0.524)	(0.526)	(0.613)	(0.607)	(0.606)	(1.166)	(0.542)
lnemploymentrate					0.0453	0.122	0.176	0.136	0.647*	0.191
					(0.192)	(0.191)	(0.188)	(0.188)	(0.362)	(0.168)
lnsalary						1.681***	1.526***	1.897***	2.523***	1.768***
						(0.484)	(0.478)	(0.501)	(0.965)	(0.448)
lnlandsalesrevenu							0.250***	0.248***	-0.155*	0.273***
							(0.0584)	(0.0582)	(0.112)	(0.052)
lnFDI								-0.117**	-0.475***	-0.108**
								(0.049)	(0.094)	(0.0438)
Cons	4.253***	6.223***	6.374***	34.82***	34.84***	29.69***	27.48***	25.80***	-14.92**	16.62***
	(0.729)	(1.506)	(1.503)	(4.935)	(4.940)	(5.109)	(5.063)	(5.089)	(9.823)	(4.552)
time effect	control	control	control	control	control	control	control	control	control	control
Regional effects	control	control	control	control	control	control	control	control	control	control
R2	0.8967	0.8971	0.8979	0.9044	0.9044	0.9065	0.9102	0.9111	0.807	0.9231
Number of samples	588	588	588	588	588	588	587	587	580	587

Standard errors in parentheses

*** p<0.01

** p<0.05

* p<0.1.

## 5.2.1 Main factors affecting value-added land dividends

The regression results in Equation (8) from Table 3 reveal a significant positive correlation between fixed asset investment and value-added land dividends in the market. On one hand, investment in fixed assets affects industrial capital from the aggregate and structure through capital accumulation and leads to industrial development and structural changes from the supply side. On the other hand, investment in fixed assets drives the rapid development of supporting industries around cities, promotes urban employment, and accelerates the development of the land economy. At the international level, there is a negative correlation between the level of regional foreign trade and the amount of value-added land dividends, indicating that trade has a certain spillover effect on the growth of value-added land dividends to a certain extent. For enterprises, the number of patent applications in each region positively influences the number of value-added land dividends. Technological innovation primarily enhances output per unit land area by generating new products, services, and technologies, thereby improving the output value per unit land of agricultural and industrial land and fostering the formation of value-added land dividends. At the social level, residents’ social welfare level exhibits a significant negative correlation with the number of value-added land dividends, with a coefficient of -4.209. This suggests that residents’ focus on social welfare and the ecological environment has diminished their pursuit of value-added land dividends, resulting in a certain crowding out effect of increased social welfare on value-added land dividends.

At the government level, there is a positive correlation between urbanization and value-added land dividends. Urbanization facilitates the optimization of the regional industrial structure, accelerates the transfer of the labor force from rural to urban areas, and speeds up the change in the amount of urban industrial construction land. For enterprises, the increase in labor wages contributes to the formation of value-added land dividends. This is because a large number of jobs are provided to attract a substantial labor force shortly after the conversion of agricultural land to industrial land, leading to fewer farmers remaining in their original agricultural positions. Land contracting enhances the efficiency of agricultural output. At the government level, land transfers have a positive impact on generating value-added land dividends. They are directly tied to local government revenue, facilitating the development of regional public services and enhancing the value of land development rights. Moreover, the government’s emphasis on infrastructure investment, coupled with the financial accelerator effect, drives the transformation of land use by local authorities.

In the short term, local governments have ensured economic growth through land finance, and value-added land dividends have increased rapidly. At the international level, the foreign investment level has a significant negative impact on value-added land dividends, indicating that external capital investment in Chinese enterprises has a relatively significant external spillover effect. FDI is the comprehensive product of multinational companies’ global strategy and the host government’s investment introduction policy. China implemented an export-oriented economic growth strategy, and the labor-intensive industry is the most exported. A large number of cheap labor forces are gathered in industrial fields, while the service industry which requires higher knowledge and technology content of labor has not developed significantly.

## 5.2.2 Main influencing factors of land efficiency dividends

As shown in the regression results of equation (9) in Table 3, fixed asset investment exhibits a positive correlation with land efficiency dividends, albeit not significantly pronounced. This suggests that appropriate and stable investment fosters the growth of land dividends during the economic take-off stage, providing essential material capital support for the structural transformation of various industries. However, as China transitions from high-speed development to high-quality development, it should facilitate capital reallocation among sectors, expedite resource flow to sectors with high labor productivity, and ensure a steady increase in efficiency dividends. The quantity of land efficiency dividends correlates positively with the level of regional trade import and export, indicating that trade activities can effectively enhance macroeconomic policies’ quality. They enable the introduction, imitation, and absorption of advanced technologies from trading countries, thereby enhancing land use efficiency through the influence on factor utilization efficiency and international competition, thus promoting the improvement of land efficiency dividends. However, the current impact is not highly significant, suggesting that the influence of trade import and export on land efficiency dividends remains somewhat limited. Technological innovation exhibits a positive correlation with land efficiency dividends, indicating its crucial role in enhancing enterprise productivity. Enterprise technological innovation serves as a key factor in driving industrial and structural transformation. Although the current effect may not be sufficiently significant, the continuous increase in land efficiency dividends reflects a gradual realization of the effects of technological innovation. There exists a positive correlation between the level of social welfare and the quantity of land efficiency dividends, suggesting that residents’ attention to welfare level brings about positive externalities. Such attention promotes enterprises’ focus on growth quality and efficiency improvement, enhancing their awareness and understanding of ecological environment quality.

At the government level, there is a significant positive correlation between urbanization and land efficiency dividends, indicating that government-led urbanization planning and construction of rural agricultural land and urban industrial construction land contribute to maintaining the original land nature, improving land use efficiency, increasing industrial economic profits, and ensuring the formation of land efficiency dividends. From a labor perspective, wage improvements attract and consolidate more high-end talents, accelerating the transition of industries from primary and secondary levels to the tertiary level, thus enhancing land use efficiency. However, land transfer behaviors restrain the growth of land efficiency dividends. This is primarily due to the fact that accelerated land circulation tends to induce short-sighted behavior among local governments in the long run, leading to issues such as redundant construction and inefficient land use, as well as stagnation and regression of regional industrial structure, which are detrimental to regional development and quality enhancement. Attracting foreign investment constitutes a crucial aspect of China’s reform and opening up. With the expansion of investment scale and enhancement of foreign-invested enterprises’ investment level, China has gained advantages in high-value-added products and technology research and development. This further promotes the improvement of efficiency dividends and the upgrading of product structure in technology and capital-intensive industries, gradually forming industries with high technology content and added value.

## 5.2.3 Main factors affecting the total amount of land dividend

The final column of data in Table 3 presents the regression outcomes of each variable on the total amount of land dividend. In general, fixed capital investment, technological innovation, urbanization, labor wages, and land transfer behaviorexhibit a positive impact on the total amount of land dividend, while trade import and export, residents’ social welfare level and foreign investment level have a negative correlation with the total amount of land dividend.

The results in Table 3 indicate a similar correlation to that of value-added land dividend, albeit with slightly different coefficients.

## 6 Conclusions and recommendations

### 6.1 Conclusions

Starting from the research on the classification, formation, calculation, and influencing factors of land dividends, the paper first expands the scope of theoretical analysis to the formation of land dividends under different land classification standards, focusing on the analysis of the formation and manifestation of value-added land dividends and land efficiency dividends, and then calculates the different types of land dividend from theoretical and empirical perspectives. It also analyzes the spatio-temporal evolution of land dividends in China and finally establishes stepwise regression models to explore the influencing factors of land economic dividends, which has important theoretical and practical significance for discussing how to continue exploring and maintaining land dividends.

Based on the preceding analysis, this paper primarily draws the following conclusions: (1) land dividends can be categorized into value-added land dividends and land efficiency dividends. (2) Value-added land dividends primarily stem from changes in land use, which in the short term lead to a rapid enhancement of economic benefits per unit area of land. Meanwhile, land efficiency dividends arise from improvements in land use efficiency while maintaining the original land use. (3) The spatio-temporal evolution study shows that the value-added land dividend generally presents a trend of first rising and then slowly declining, and the change is most obvious in the central region. This is due to the decrease in land quantity caused by the change of use, and the land efficiency dividend presents a fluctuating upward trend. Similar to the value-added land dividend, the total amount of land dividend typically follows a pattern of initial growth followed by a gradual decline. (4) Research on influencing factors shows that the value-added land dividend and the total land dividend are significantly positively correlated with land granting, capital input, and other factors, and the land efficiency dividend is significantly positively correlated with urbanization and labor wages.

### 6.2 Recommendations

1. Rationally regulating the use of land indicators and spatial layout is an important way of promoting sustainable urban development. With regard to the use of land indicators, emphasis should be placed on the allocation of land elements at the stage of urban master planning on the basis of extensive territorial spatial planning, in which the actual needs and potential growth of urban development need to be comprehensively assessed to ensure that the allocation of land indicators meets the goals of long-term urban development, while attention should also be paid to avoiding the wasteful and inefficient use of land resources. The existing dual structure of urban and rural land allocation should be broken down in terms of spatial layout, and the pattern of resource flows between urban and rural areas should be promoted so as to gradually form integrated urban and rural development, which will not only improve the efficiency of land use, but also promote synergistic development of the urban and rural economies, guaranteeing a strong increase in the land dividend.
2. Optimized Land Utilization. To accelerate the transition from land value-added dividends to land efficiency dividends, the government must strictly enforce land audit thresholds and regulate the use of urban and rural land. Additionally, it should further reduce land acquisitions through alternative means, expand the coverage of bidding and auction methods, and enhance the efficiency of land resource utilization. In line with local ecological red lines and environmental protection requirements, efforts should be made to revitalize inefficient, idle, and semi-developed land, while promoting the standardization, intensification, and conservation of land use. Furthermore, by aligning with policies that link the increase and decrease of urban and rural construction land, the transformation of rural construction land into urban construction land should be encouraged, alongside the regulation of arable land resources, to promote their efficient use.
3. Stimulating High-Quality Capital Investment and Talent Inflow. Encouraging various forms of capital inflow into the market and providing long-term interest discount support for enterprises is crucial. The government should establish an institutional environment conducive to promoting urban land investment and ensuring long-term economic stability. Moreover, rational guidance of labor flow between regions, coupled with enhanced education and skill training for rural labor, will boost farmers’ capabilities and competitiveness in non-agricultural sectors.
4. Leveraging Ecological Compensation and Social Security Functions. Utilizing fiscal transfers to augment investment in ecological compensation and farmers’ social welfare is paramount. Establishing multiple compensation mechanisms for ecological protection, such as compensation for land resources and development, will enhance the market-oriented rural land system and safeguard the livelihoods of landless farmers. Ensuring the rational and lawful transfer of rural land, fostering steady rural economic growth, and improving the rural ecological environment are essential government responsibilities.
